# Interactions of the DNA Nanostructure with Silane-Based Self-Assembled Monolayers

**DOI:** 10.1021/acs.langmuir.5c06157

**Published:** 2026-03-30

**Authors:** Shubhankar Kundu, Sydney P. Moore, Anumita Kumari, Haitao Liu

**Affiliations:** Department of Chemistry, 6614 University of Pittsburgh, Pittsburgh, Pennsylvania 15260, United States

**Keywords:** DNA nanostructures, self-assembled monolayer (SAM), hydrophobicity, water contact angle, surface

## Abstract

We show that the interactions with surfaces have a significant effect on the morphological outcome of deposited DNA nanostructures. We have modified silicon oxide surface with single and mixed self-assembled monolayers (SAMs). Amine-terminated silane was mixed with alkyl chlorosilanes of different chain lengths (C_18_, C_6_, and C_4_) for SAM preparation to modulate the polarity and therefore wettability of the surface. We deposited DNA nanostructures onto these SAM-modified surfaces. Increasing the hydrophobicity of the surface increases the density of the deposited DNA nanostructure but at the cost of severe structural deformation. On the other hand, the number density of nondeformed DNA nanostructure initially increases and then decreases with increasing hydrophobicity of the surface.

## Graphical abstract

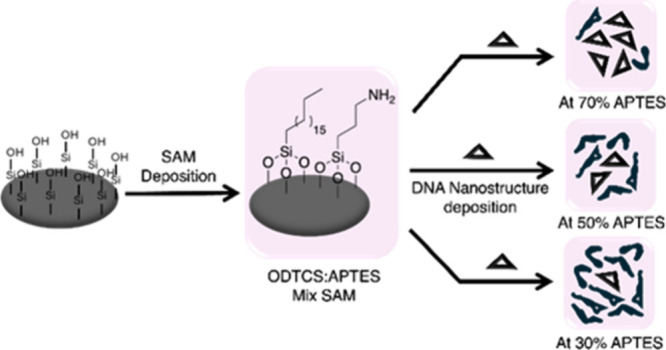

## Introduction

The concept of using the origami approach to create nanostructures with DNA was first demonstrated by Rothemund in 2006 to generate a variety of complex shapes.^1,2^ DNA nanotechnology has since found promising application in biosensing,^3^ drug delivery,^4,5^ templated material synthesis,^6^ nanophotonics,^7–10^ nanoelectronics,^11^ plasmonics^7^ and nanorobotics.^12–15^ Thus, despite its relatively recent emergence in the scientific community, the field offers significant potential for future exploration and innovation. A lot of the applications of the DNA nanostructure require proper understanding of their structural and functional stability as well as integrity after being deposited on surfaces.^2,16^ Therefore, it is very important to address the effect of the surrounding environment on the stability of DNA nanostructures.

Many applications of the DNA nanostructure require a supporting substrate. However, not all substrates are compatible with DNA nanostructures because they are extremely sensitive to environmental factors, such as pH, surface tension, and the chemical nature of the substrate.^17,18^ DNA nanostructures, being amphiphilic in nature, are stable when adsorbed or deposited at hydrophilic surfaces such as mica and silicon dioxide (SiO_2_)/Si wafer or on charged surfaces via ionic interactions.^19,20^ Having stable DNA nanostructures on these substrates allowed researchers to develop applications, such as DNA-based lithography of SiO_2_.^21,22^

In contrast, deposition of DNA nanostructures on hydrophobic substrates is much less explored. Hui et al. reported that the DNA nanostructure could be deposited onto polystyrene substrate in the presence of 1-pyrenemethylamine hydrochloride (PMA) coating, which helps to stabilize the nanostructures on the surface via the positive charges of the amine groups. Absence of PMA pretreatment on the substrate results in severe deformation of the nanostructures.^20^ Research has also been done on the interaction between DNA nanostructures and 2D surfaces like graphene^23^ and highly oriented pyrolytic graphite (HOPG).^24^ PMA coating was required to successfuly deposit DNA nanostructures onto graphene; on HOPG, the DNA nanostructures were significantly deformed without PMA coating, even though the overall shapes were preserved. It was hypothesized that the π−π stacking interaction of aromatic groups present in DNA nanostructures, with the surface, caused their deformation on the graphitic surface. Similarly, pristine molybdenum disulfide (MoS_2_) surface deformed the nanostructure as well but having a PMA coating on it resulted in successful deposition without structural deformation.^25^

We are interested in a systematic understanding of the interaction between the DNA nanostructure and hydrophobic substrates. Such an understanding is critical to a broader range of future applications of DNA nanostructures. In this study, we chose SAM as our model system to probe the role of wettability in the outcome of the deposition. Silane-based SAMs are known for their versatility in modifying a wide range of surfaces, their ability to tune surface properties such as wettability, and accessibility for grafting with different chemical groups to develop biosensors,^26,27^ microelectrodes,^28^ etc. SAMs have well-defined surface morphology and very low surface roughness, which are important for characterization of deposited DNA nanostructures. Moreover, silane-based SAMs are exceptionally stable, attributed to the strong covalent bonding between the SiO_2_ surface and the silanes.^29^ SAM-based modification allows for easy manipulation of the surface chemistry by adjusting the chemical composition of the silanes or making further chemical transformations on the newly formed monolayers. This approach enhances the versatility and usability of the surfaces, making it a popular choice for introducing surface modifications.^29,30^

Our group has recently studied the deposition of DNA nanostructure on several single-component SAMs, including aminopropyltriethoxysilane (APTES), phenyltrichlorosilane (PTCS),^31^ phenylhexyltrichlorosilane (PHTCS), and octadecyltrichlorosilane (ODTCS).^32,33^ These results showed that SAM has a significant impact on the stability of the DNA nanostructure. Hydrophobic single-component SAMs like ODTCS deform the deposited nanostructures, whereas more hydrophilic SAMs, such as amine-terminated APTES^34,35^ or phenyl terminated PTCS, allow the DNA nanostructure to maintain the structural stability. This work indicated that the stability of DNA nanostructures is influenced by multiple factors, including ionic interactions with surface functional groups (*e.g.,* primary amine), π−π stacking (*e.g.,* phenyl ring), and hydrophobicity of the surface (*e.g.,* long chain alkyl).^33^

**Scheme 1:**
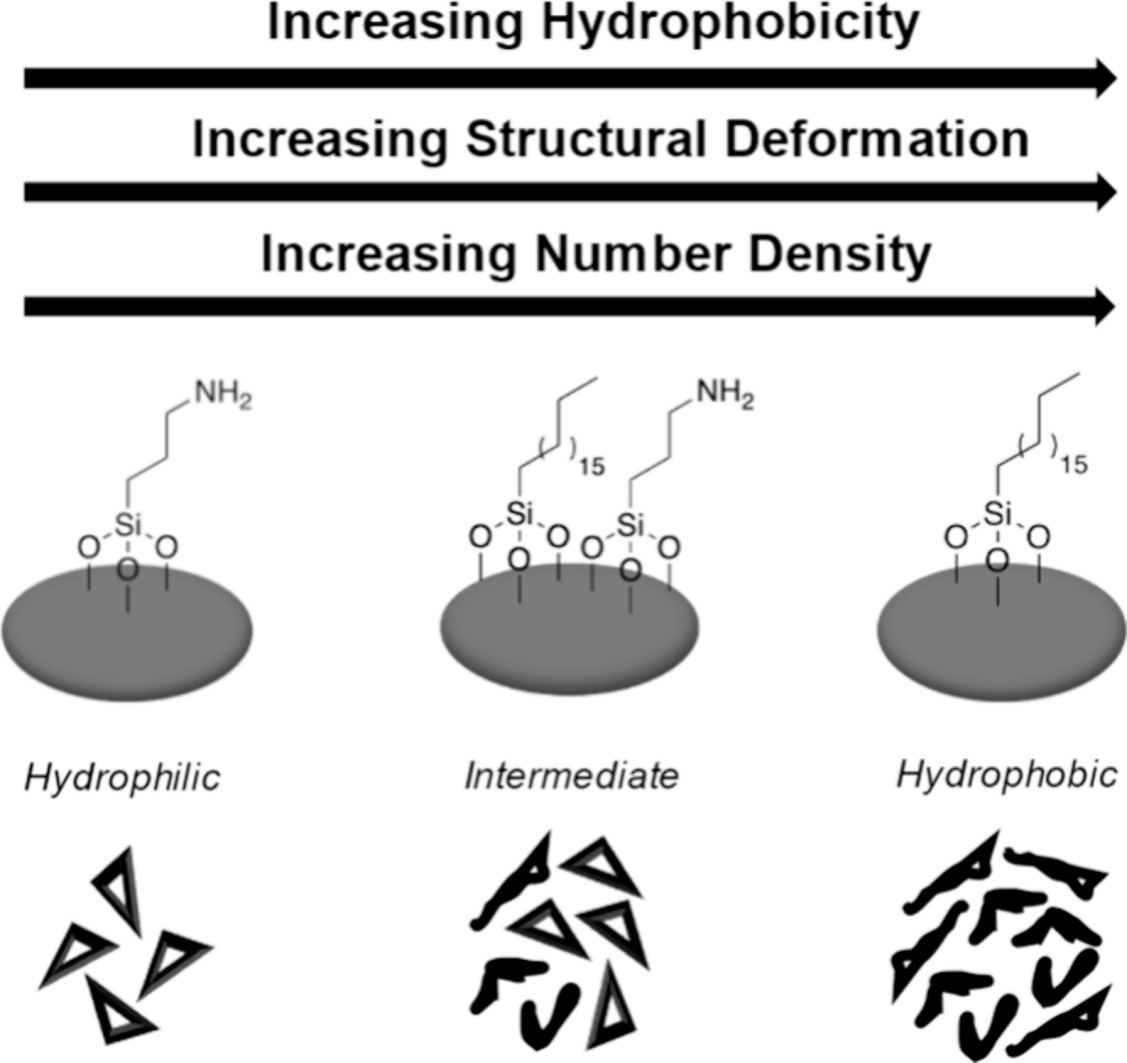
Morphology of the DNA Nanostructures upon Deposition onto Different SAMs

In this paper, we report a systematic study using a two-component SAM to elucidate the role of wettability and chemical composition on the structural integrity of the deposited DNA nanostructure. As shown in Scheme 1, we prepared mixed-silane SAMs with very different polarities by adjusting themolar ratios of the silane precursors in the solvent (*e.g.*, 3:7, 5:5, and 7:3). We observed a strong dependence of the number density and structural stability of DNA nanostructures on the SAM composition. This study will advance our fundamental understanding of DNA nanostructure−surface interactions and will aid the future integration of DNA nanotechnology with SAM-based applications like protein adsorptions,^36–40^ cell adhesions,^37,41^ bioactive surfaces,^42–44^ biosensors,^26,27^ microelectronics,^45^ and optoelectronics.^46^

## Experimental Section

### Materials

Silicon wafers [100], coated with native oxide layers, were procured from University Wafers and WaferPro. The M13mp18 scaffold and synthetic staple DNA strands utilized for fabricating the DNA nanostructures were sourced from New England Biolabs and Integrated DNA Technologies (IDT), respectively. Chemical reagents were used as received. Magnesium acetate tetrahydrate, sulfuric acid, hydrogen peroxide solution (30% H_2_O_2_), sodium chloride (≥99.0%), ethanol, hexane (mixture of isomers, ≥ 98.5%), and toluene (≥99.5%) were acquired from Sigma-Aldrich. Hydrochloric acid and sodium hydroxide were obtained from Fisher Scientific. The silanes used in this study include APTES (Thermo Fisher Scientific), butyltrichlorosilane (BTCS, Sigma-Aldrich), trichlorohexylsialne (HTCS, TCI Chemicals), and ODTCS (Acros Organics). Water (18.3 MΩ) was purified using a water purification system (Barnstead Easypure II, Thermo Scientific, Waltham, Massachusetts).

### Methods

#### Preparation of SAMs

Prior to the preparation of SAM, all glassware underwent thorough rinsing with hexane, acetone, and DI water, followed by drying in an oven at approximately 180 °C for 24 h. New 20 mL disposable scintillation glass vials and their caps were obtained from Kimble Glass, Inc. Tweezers were rinsed successively with hexane, acetone, 2-propanol, and DI water and then dried with strong N_2_ flow. Si[100] wafers were cleaned in piranha solution (H_2_SO_4_ (98%)/H_2_O_2_ (30%), 70:30 (v/v)) at 85 °C for 45 min. *Warning: Piranha solution is a strong oxidizing reagent and can explode unexpectedly when in contact with organic materials. Extra caution in handling is required.* The cleaned wafers were rinsed with water and dried with N_2_ until no water drops were visible. Water contact angle (WCA) of the cleaned Si wafer was 4 ± 1°. Single-component and mixed SAMs of ODTCS, APTES, HTCS, and BTCS were prepared at room temperature inside a glovebox. More details about the SAM preparation are provided in the Supporting Information.

#### Ellipsometry Measurement of SAMs

Thickness of the native oxide and the added SAM layer were measured using a J.A. Woollam Co. alpha-SE ellipsometer in the wavelength range of 380−900 nm with an angle offset of 70°. Experimental details of the thickness measurement are discussed in the Supporting Information.

#### Characterization of SAMs Using WCA Measurements

Surface energy and WCA measurements were performed using a VCA Optima XE instrument under ambient conditions of temperature (22−25 °C) and relative humidity (20−40%). In short, 1 μL liquid droplets were formed and drop-casted onto the surface at different spots to get an average value of WCA in one trail. Reported values represent the average of three to four repeated trails. Please refer to the Supporting Information and Figure S1 for more details.

#### X-ray Photoelectron Spectroscopy (XPS) Analysis of SAMs

XPS measurements for SAM characterization were carried out by using a Thermo Scientific Escalab 250 Xi instrument. The X-ray source utilized a monochromatic Al anode with a spot size of 0.2 mm for the SAM, set at a takeoff angle of 45°. Experimental details about scanning and analysis of both survey spectra as well as high-resolution spectra (Figures S2 and S3) are discussed in the Supporting Information.

#### Imaging SAMs Using Atomic Force Microscopy (AFM)

The morphology and height of the nanoscale patterns were characterized by an Asylum MFP3D AFM in AC-air tapping mode using HQ/NSC15/AlBS AFM probes (325 kHz, 40 N/m) purchased from μmasch (NanoAndMore USA). All images were collected at a scan rate of 1.0 Hz and 512 data points per line.

A detailed account of methods and experimental procedures can be found in the Supporting Information.

## Results and Discussion

### Preparation and Characterization of SAMs

We prepared single and multicomponent SAMs using APTES, BTCS, HTCS, and ODTCS. Preparation of single-component SAMs was carried out using a solution-based procedure following a previously reported method.^32,47,48^ ODTCS/APTES mixed SAMs were prepared by exposing Si wafers to a mixture of the two silanes. For mixed APTES and BTCS/HTCS, we first exposed the wafer to pure BTCS/HTCS solutions, followed by immersing the wafer in pure APTES solution. In all of these experiments, we changed the molar ratios of the silanes in solution (7:3, 5:5, and 3:7) to tune the composition of the mixed SAMs. Characterization of SAM samples was accomplished through XPS, WCA measurements, and ellipsometry.

XPS data indicate successful variation of the SAM composition. To quantify the composition of the SAMs, we used the areas of the N 1s peak for the primary amine in APTES (binding energy: 399 eV) and the Si 2p peak for the bulk Si wafer (binding energy: 96 eV). The N/Si ratio, derived from the ratio of the peak area, is shown in Table 1 and Figures S2 and S3. We conducted a similar analysis using the C 1s peak (284 eV) to obtain the C/Si ratio. However, we found that the carbon content is very sensitive to hydrocarbon contamination within the instrument, making it less reliable to analyze the surface composition. As can be seen in Table 1, the N/Si (N:Si) ratio increased when higher concentration of APTES was used in preparing the mixed SAMs.

**Table 1:** Intensity Ratio of N 1s versus Bulk Si 2p XPS Signal

SAM	N 1s/Si 2p^a^			
ratio^b^	0:1	3:7	5:5	7:3
ODTCS:APTES	0.114	0.263	0.165	0.102
HTCS:APTES	0.114	0.108	0.073	0.051
BTCS:APTES	0.114	0.124	0.095	0.056

a XPS recorded 12 h after preparation.

b Molar ratio of silanes in solvent.

We measured the film thickness of the SAM samples using ellipsometry, shown in Table 2. For the single-component SAM samples, our data are reasonably consistent with reported values (*e.g.*, for HTCS, we measured 1.3 nm vs reported 1.2 nm). For the mixed SAMs, we do not observe a clear trend in the thickness and there is no literature data available to compare to. The thickness of SAM is likely a function of the packing and tilting of the silane molecules, both of which could vary as a function of the chemical structure of the silanes. For example, longer alkyl silanes like ODTCS are reported to pack well during monolayer deposition, resulting in superior SAM quality compared to shorter alkyl chains like BTCS and HTCS.^47^ The thickness data here suggest a reasonable agreement with the monolayer structure.

**Table 2:** Ellipsometry Height of the SAMs

SAM	height (nm)^a^				
ratio^b^	0:1	3:7	5:5	7:3	1:0
ODTCS:APTES	0.5 ± 0.3	1.7 ± 0.7	1.7 ± 0.3	1.7 ± 0.2	2.6 ± 0.4
HTCS:APTES	0.5 ± 0.3	1.4 ± 0.4	1.2 ± 0.3	1.9 ± 0.9	1.2 ± 0.2
BTCS:APTES	0.5 ± 0.3	1.6 ± 0.6	1.4 ± 0.4	1.3 ± 0.4	0.9 ± 0.2

a Ellipsometry measurement of the mixed SAMs 12 h after preparation.

b Molar ratio of the silanes in solvent.

WCA measurement provided additional insight into the composition of the mixed SAMs. As shown in Table 3, for the ODTCS:APTES samples, we observed a clear transition as a function of the surface composition. However, for HTCS and BTCS mixed SAMs, all samples show a very similar WCA, despite the different nitrogen content reported by XPS. Given our interest in understanding the effect of wettability on the structural integrity of DNA nanostructures, we focused on the ODTCS: APTES mixed SAM in our studies below.

**Table 3:** WCA of SAM

SAM	WCA (degrees/°)^a^				
ratio^b^	0:1	3:7	5:5	7:3	1:0
ODTCS:APTES	67 ± 2	80 ± 2	90 ± 3	95 ± 1	100 ± 1
HTCS:APTES	67 ± 2	94 ± 2	91 ± 3	91 ± 3	99 ± 4
BTCS:APTES	67 ± 2	89 ± 5	90 ± 4	93 ± 3	94 ± 4

a Water contact angle of the mixed SAMs after 12 h of preparation.

b Molar ratio of the silanes in solvent.

For the ODTCS:APTES mixed SAMs, we have calculated surface energy using contact angles of diiodomethane and water. Figure S1 shows the surface energy of the SAMs as a function of the ODTCS:APTES concentration ratios. As expected, APTES SAM, having polar amine termination, has the highest surface energy and the lowest WCA, whereas increasing the percentage of the ODTCS in the multicomponent SAMs results in decreasing surface energy and increasing the WCA value.

### Correlation of Wettability with Structural Integrity of DNA Nanostructures

We deposited DNA triangle nanostructures onto the mixed-SAM substrates from a pH 8 buffer and characterized their morphology using AFM. Previous work on single-component SAM showed that the stability of DNA nanostructures is dependent on the chemical composition of the SAM. In this work, we observed a strong dependence on the mixed SAM composition as well (Figures 1, S4−S7).

**Figure 1:**
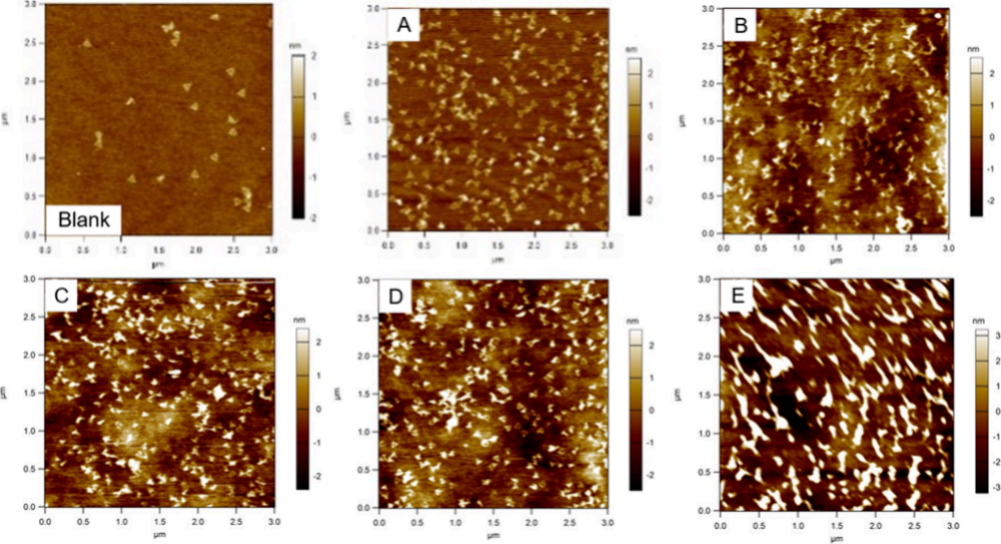
AFM images of APTES:ODTCS mixed SAMs with ratios. (A) 1:0, (B) 7:3, (C) 5:5, (D) 3:7, (E) 0:1 (*blank: silicon wafer*).

AFM images in Figure 1 (see Figure S7 for an enlarged view and Figure S8 for data from a separate set of experiments) show the outcome of nanostructure interaction with a SAM-modified surface containing ODTCS:APTES at different ratios. The DNA nanostructures maintained their structural integrity on the APTES SAM (Figure 1A). Increasing percentage of the nonpolar ODTCS in the mixed SAM results in increasing structural deformation (Figure 1B−D). All the DNA triangles are deformed when deposited onto a single component ODTCS SAM (Figure 1E).

The composition of the mixed SAM also greatly impacts the number density of the DNA nanostructures deposited on the substrate. Figure 2 shows the total number of DNA nanostructures deposited on the ODTCS:APTES SAM samples, and the error bars shown have been calculated from the replicates of the respective substrates. Several features are worth commenting on. First, the number density of completely deformed and partially deformed DNA triangles increased with an increasing fraction of ODTCS in the SAM. However, the number density of the structurally intact DNA nanostructures does not follow a monotonic trend and instead peaked at the 3:7 ODTCS:APTES ratio. To further probe the underlying mechanism of such behavior, we also plot the total number density and percentage of intact DNA nanostructures (%T) as a function of the surface energy (Figure 3 and Table S1). As can be seen, lowering the surface energy (i.e., higher contact angle) increases the total number density of DNA nanostructures on the surface but decreases the percentage of intact DNA nanostructures. It is interesting to note that the percentage of intact DNA nanostructure decreases almost linearly with decreasing surface energy while the change in number density is very abrupt at both ends of the surface energy range.

**Figure 2:**
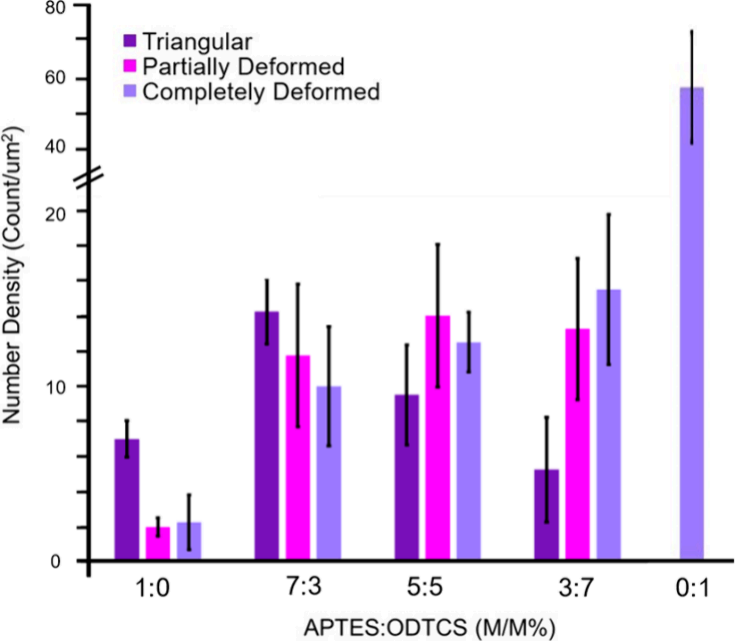
Number of DNA nanostructures on APTES:ODTCS mixed SAM (per μm^2^) at different compositions.

**Figure 3:**
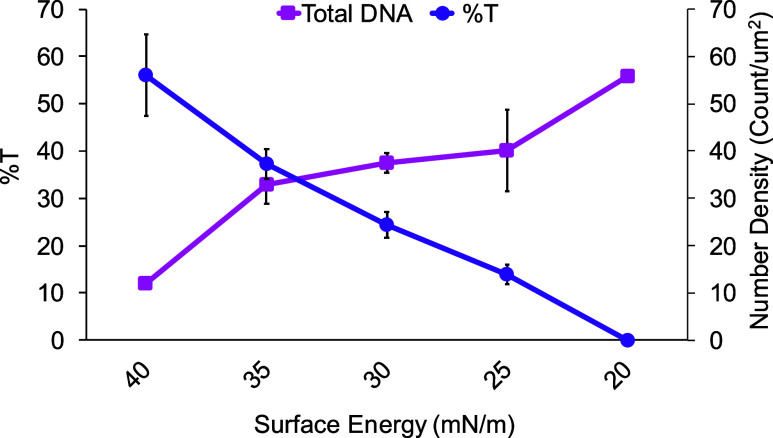
Comparison of number density versus percentage of intact DNA triangles (%*T*) at different surface energies.

We have conducted similar experiments on SAMs prepared using BTCS/HTCS and APTES. Single-component BTCS or HTCS SAM resulted in complete deformation of the triangles similar to those observed on the ODTCS SAM, albeit with much lower number density of nanostructures (Figure S4). However, in all the mixed SAMs of APTES with BTCS (Figure S5) or HTCS (Figure S6), we did not observe a significant fraction of intact DNA nanostructures. In addition, we also found that the outcome of DNA deposition on these mixed SAMs is less reproducible. We speculate that this poor reproducibility may be attributed to the small interchain packing interaction and difficulty in forming good-quality monolayers. Table S2 summarizes the morphology of the DNA nanostructures upon deposition onto all of the SAM samples.

To understand our data, we consider two factors impacting the morphology of the DNA nanostructure: stability of the double helix structure and interfacial force experienced during the drying process. As shown in Scheme 2, electrostatic interactions between positively charged ions (*e.g.*, APTES in our experiment) and the negatively charged phosphate backbone of the DNA stabilize the double helix structure. On the other hand, there is a hydrophobic effect between the DNA bases and the alkyl chains in the SAM and such interactions would destabilize the double helix. In our experiment, the number density of the DNA nanostructure increases with increasing fraction of the ODTCS in the SAM, suggesting the hydrophobic effect being stronger than electrostatics. However, the number densities of DNA nanostructures are very different between single-component ODTCS and BTCS/HTCS SAM samples, even though they have a similar wettability. Thus, the chain length of the SAM may also contribute to the overall magnitude of the hydrophobic effect.

**Scheme 2:**
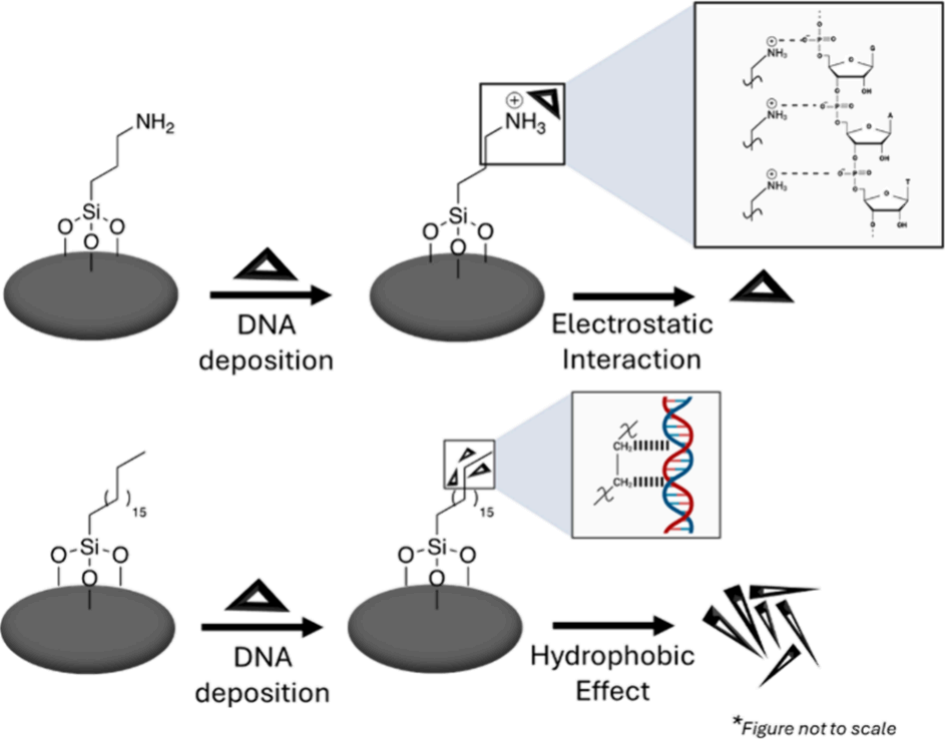
Interaction of Nanostructures with the Surface

**Scheme 3:**
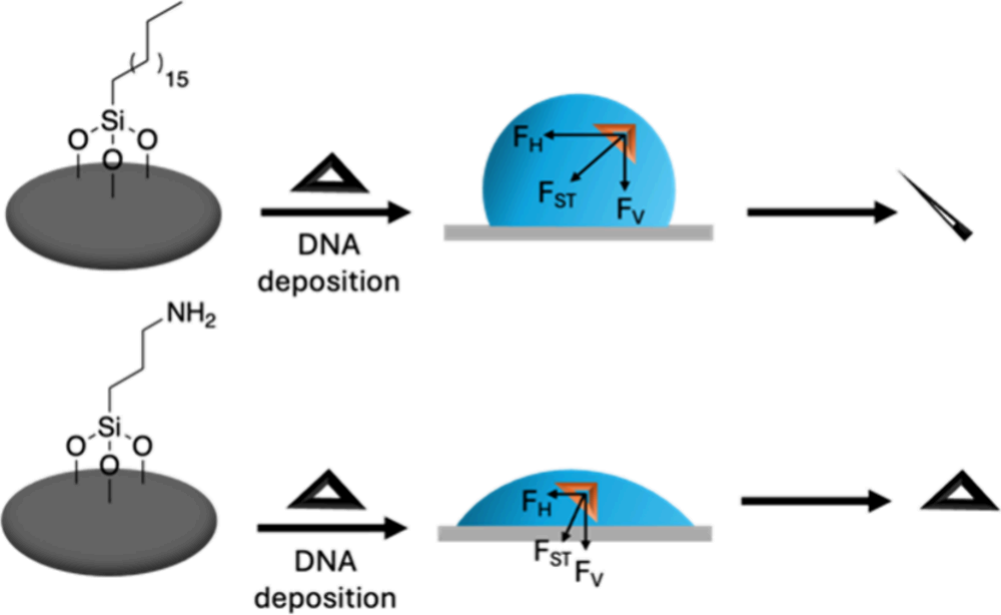
Effect of Wettability on DNA Nanostructure Stabilization

DNA nanostructures also experience surface tension during the drying process. As shown in Scheme 3, surface tension, *F*_ST_, experienced by the deposited nanostructures during the solvent drying process has two components such as *F*_H_ (horizontal) and *F*_V_ (vertical). The horizontal component, *F*_H_, is larger for a more hydrophobic surface (up to a contact angle of 90°) and more likely to deform the DNA nanostructures.

## Conclusions

We prepared mixed SAMs using APTES and three alkyl silanes (ODTCS, HTCS, and BTCS) to study their interaction with DNA nanostructures. The morphology and number density of deposited DNA nanostructures are sensitive to the composition of the mixed SAM. We found that the surface with a higher concentration of the polar amine-terminated silane mixed with the long-chain nonpolar alkyl chain (C_18_) (i.e., 3:7 mol/mol % ODTCS:APTES in solvent) resulted in a maximum density of deposited DNA nanostructures with structural integrity. It is likely that the degree of deformation also depends on the mechanical strength of the nanostructures, and work is underway to fully understand the dynamics of solvent drying on the outcome of the deposition of DNA nanostructures. This work provides insight into the interaction between DNA nanostructures with SAM-modified surfaces and provides guidelines to optimize the outcome of the deposition of DNA nanostructures. We hope that it will broaden the application window of DNA nanotechnology to advance material science and nanomedicine research.

## Supplementary Material



## Notes

Experimental details, additional AFM images, contact angle, and surface energy data (Figures S1−S8 and Tables S1−S2) (PDF)
