# Supplementary material for: Interactions of the DNA Nanostructure with Silane-Based Self-Assembled Monolayers
Source: Langmuir. 2026 Mar 30;42(14):9754–9. doi: 10.1021/acs.langmuir.5c06157 (PMC13085813; doi:10.1021/acs.langmuir.5c06157)
Supplement: Supplementary file 1 [file la5c06157_si_001.pdf]

Interactions of DNA Nanostructure with Silane-Based Self-Assembled Monolayers

Shubhankar Kundu, Sydney P. Moore, Anumita Kumari, Haitao Liu*

Department of Chemistry, University of Pittsburgh, Pittsburgh, PA, 15260, USA

KEYWORDS. DNA nanostructures, Self-Assembled Monolayer (SAM), Hydrophobicity, Water Contact Angle, Surface

Supporting Information

Table of Contents

1. Materials and Instruments
2. Methods
   a) Preparation of buffer solution
   b) Synthesis of DNA nanostructures
   c) Preparation of Self-Assembled Monolayers (SAMs)
   d) Characterization of SAMs using ellipsometer
   e) Characterization of SAMs using Contact Angle (CA) measurements
   f) Characterization of SAMs using X-ray Photoelectron Spectroscopy (XPS)
   g) Deposition of DNA nanostructures onto SAMs
   h) Imaging the SAMs using Atomic Force Microscopy (AFM)
   i) XPS characterization data of SAMs
3. AFM images of the deposited nanostructures
4. Morphology and number density study of the nanostructures deposited on SAMs
5. References

1. Materials and Instruments

Silicon wafers [100], coated with native oxide layers, were procured from University Wafers and WAFERPRO. The M13mp18 scaffold and synthetic staple DNA strands utilized for fabricating the DNA nanostructures were sourced from New England

Biolabs and Integrated DNA Technologies (IDT), respectively. Chemical reagents including magnesium acetate tetrahydrate, sulfuric acid, hydrogen peroxide solution (30% H_2O_2), sodium chloride (≥99.0%), ethanol, hexane (mixture of isomers, ≥98.5%), and toluene (≥99.5%) were acquired from Sigma-Aldrich (St. Louis, MO). Hydrochloric acid and sodium hydroxide were obtained from Fisher Scientific (Fair Lawn, NJ). We have ordered Aminopropyltriethoxysilane (APTES) from Thermo Fisher Scientific; Butyltrichlorosilane (BTCS) from Sigma Aldrich, Trichlorohexylsialne (HTCS) from TCI Chemicals and Octadecyltriclorosilane (ODTCS) from Acros Organics. Water (18.3 MΩ) was purified using a water purification system (Barnstead EASYpure II or Thermo Scientific, Waltham, MA) and employed throughout the entirety of the experiment

2. Methods

a) Preparation of buffer solution

TAE buffer (1x) was prepared by diluting commercially available 50X TAE (Tris-Acetate-EDTA) electrophoresis buffer (Thermo Fisher Scientific) with deionized (DI) water. Subsequently, magnesium acetate tetrahydrate was added to the prepared 1x buffer to achieve final [Mg^{2+}] of 12.5 mM. The pH of the buffer solution (8.0-8.3) was confirmed using both pH paper and a pH meter prior to their use in the experiment.

b) Synthesis of DNA nanostructures

The M13mp18 scaffold DNA strand solution (8.6 μL, 1.6 nM) was combined with 15 μL of the designated set of 253 staple DNA strands solution (16 nM), along with 77 μL of deionized water and 181 μL of the buffer solution (prepared at pH 8). Subsequently, the mixed DNA solution underwent annealing and filtration procedures following a previously established protocol as described in our published procedure.[S1, S2]

c) Preparation of self-assembled monolayers (SAMs)

Prior to the preparation of SAM, all glassware underwent thorough rinsing with hexane, acetone, and DI water, followed by drying in an oven at approximately 180 °C for 24 hours. New 20 mL disposable scintillation glass vials and their caps were procured from Kimble Glass, Inc. Tweezers were rinsed successively with hexane, acetone, 2-propanol, and DI water, then dried with strong N_2 flow. Si [100] wafers were cleaned in piranha solution (H_2SO_4 (98%)/H_2O_2 (30%), 70:30 (v/v)) at 85 °C for 45 minutes. *Warning: Piranha solution is a strong oxidizing reagent and can explode unexpectedly. Extra caution in handling is required.* The cleaned wafers were rinsed with water and dried with N_2 until no water drops were visible. WCA of this treated Si-wafer showed 4±1°. The clean oxidized Si [100] substrates were then immersed into freshly prepared 1×10^{-3} mol/L

solution of silane precursor with anhydrous HPLC graded hexane at room temperature inside a glovebox. For the preparation of single components and mixed SAMs, ODTCS,[S3-S5] HTCS, BTCS and APTES were used. All the mixed SAMs (*e.g.,* ODTCS: APTES, HTCS: APTES, BTCS: APTES) were prepared at three different molar ratios of the silanes (*e.g.,* 3:7. 5:5, 7:3) in the solvent. The reaction time was 2 hours for single component ODTCS as well as mixed ODTCS: APTES SAMs. For single component BTCS and HTCS silanes, deposition time was 16 hours and 4 hours respectively. For mixed BTCS/APTES and HTCS/APTES SAM samples, the substrate was exposed to BTCS or HTCS solution first, for 3.5 hours and 4 hours respectively; then the substrate was rinsed with hexane or toluene (2 mL solvent per rinse, for a total of 3 times) and immersed in a solution of APTES in a separate vial for 30 mins. Finally, substrates with mixed SAMs were cleaned again with 2 mL solvent per rinse, for a total of 3 times after APTES deposition. Reaction time for preparing single component APTES SAM was 30 min. All prepared SAMs were sonicated in acetone, chloroform, and 2-propanol for 30 minutes each to remove excess silane precursors, followed by drying under nitrogen gas stream.

d) Characterization of SAMs using ellipsometer

The thickness of the native oxide and SAM layers was measured using a J.A. Woollam Co. Alpha-SE Ellipsometer at a wavelength range of 380-900 nm with an angle offset of 70°, on the bare activated baked Si [100] wafer. Optical constants for both Si bulk substrate and SiO_2 are known. Models with these optical constants are supplied with the CompleteEASE software which we used, are named as "Si with Native Oxide" (SIO2_JAW) and "Fused silica" (SI_JAW). Both models were used for the thickness measurement of the native silicon oxide. Following SAM deposition on the wafer, the height was reanalyzed using the 'Cauchy_ZDOL (A = 1.30)' model, which provided the height of the added SAM layer on the native oxide surface.

e) Characterization of SAMs using water contact angle (WCA) measurements

WCA measurements were performed using a VCA Optima XE instrument under ambient conditions of temperature (22–25 °C) and relative humidity (20–40%). To prevent cross-contamination, a dedicated testing syringe was employed for each test liquid (e.g., DI water, Iodomethane). A liquid droplet of 1 μL was carefully formed at the syringe tip and deposited onto the sample surface. Subsequently, the syringe was withdrawn, and a static contact angle image was captured within 3 seconds of liquid deposition using a charge-coupled device camera. Contact angle values were calculated using software provided by the vendor, and reported values represent the average of 3-4 repeats. We have used WCA data to calculate surface energy (**Figure S1**) of the mixed APTES: ODTCS SAM surfaces using Owens-Wendt-Rabel & Kaelble (OWRK) model. [S6, S7]

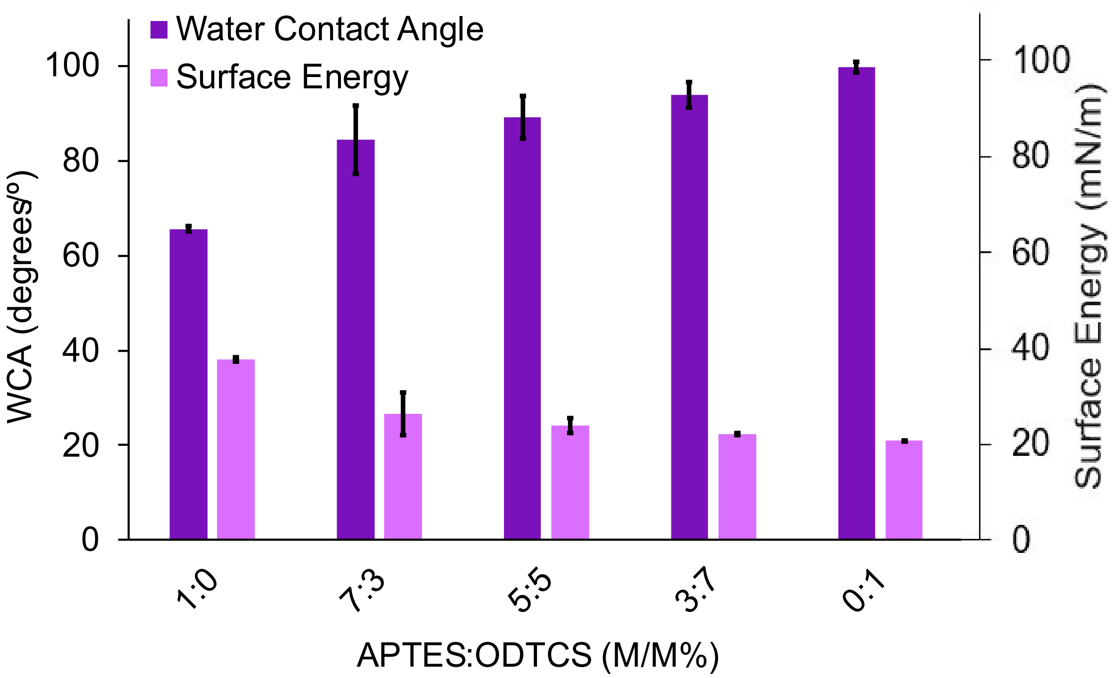

Figure S1. WCA and surface energy of APTES: ODTCS SAMs as a function of their composition.

f) Characterization of SAMs using X-Ray photoelectron spectroscopy (XPS) measurements

XPS measurements for SAM characterization were carried out using a Thermo Scientific Escalab 250 Xi instrument. The X-ray source utilized a monochromatic Al anode with a spot size of 0.2 mm for the SAM, set at a takeoff angle of 45°. A minimum of 3 survey scans were conducted to ensure a good signal-to-noise ratio, followed by higher resolution scans consisting of a minimum of 64 scans. Data acquisition, peak deconvolution, and analysis were performed using either the Thermo Scientific

Avantage Data System or the XPSPEAK 4.1 software. Peak fitting techniques enabled control over Lorentzian–Gaussian ratio and optimization of difference spectra, with the Smart method utilized for calculating the background spectrum.

g) Deposition of DNA nanostructures onto SAMs

A DNA origami solution (3 µg/mL, 100 µL) was prepared by diluting from the stock solution with 12.5 mM Mg-TAE buffer (pH 8). The solution (20 µL) was pipetted onto a Si [100] wafer coated with SAM and allowed to stand undisturbed for 30 minutes inside a Petri dish. The dish was covered with a moist Kimwipes™ paper lid to minimize evaporation of the solution. Subsequently, the substrate was dried using N_2 gas, followed by immersion in ethanol/water (90:10 v/v) solution for 15 seconds to eliminate remaining salt impurities on the surface. Finally, the substrate was dried using N_2 gas.

h) Imaging the SAMs using atomic force microscope (AFM)

The morphology and height of the nanoscale patterns were characterized by Asylum MFP3D AFM in AC-air tapping mode and HQ/NSC15/AlBS AFM probes (325 kHz, 40 N/m) purchased from µmasch (NanoAndMore USA). All images were collected at a scan rate of 1.0 Hz and 512 data points per line. The images were analyzed with Igor Pro 6.37. Typical paraemeters are: scan size 2-3 µm, scan rate 0.99 Hz, amplitude set point 250 mV, drive frequency 310 kHz and drive amplitude 47 mV. Number density for each sample was averaged from the five sets of AFM data and standard deviation was calculated from three separate sets of experiments each conducted by a different researcher.

i) XPS characterization data of SAMs

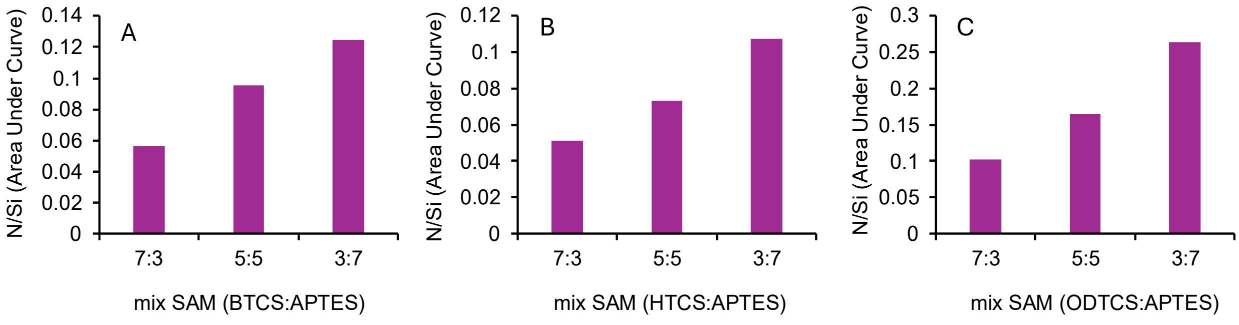

Figure S2. N1s/ Si2p ratio of the mixed SAMs

XPS was performed to confirm the composition of single-component and mixed SAM-modified surfaces. **Figure S2** shows the N1s/Si2p ratio as measured by XPS for the three types of mixed SAMs. **Figure S3** shows the N1s and Si2p signals of the XPS spectra for ODTCS: APTES mixed SAMs prepared using three different precursor ratios.

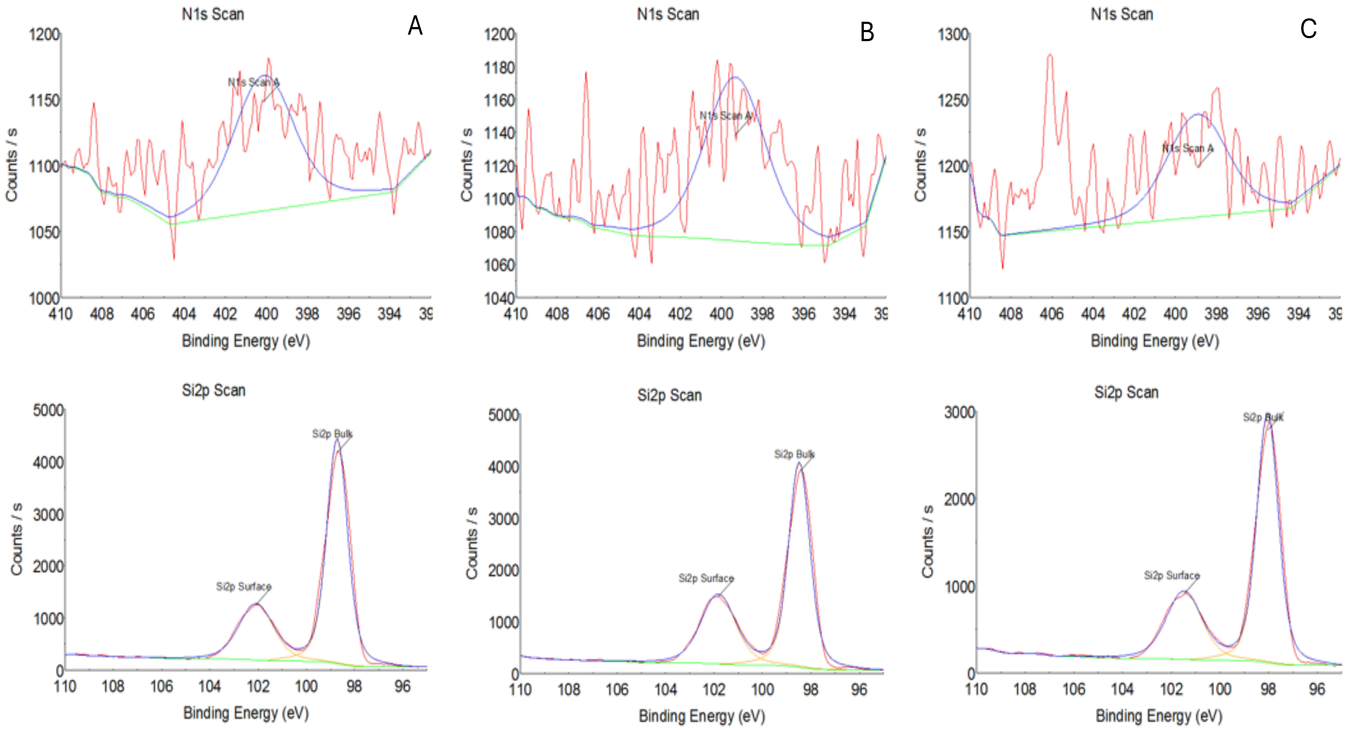

Figure S3. N1s and Si2p signals in XPS for the mixed APTES: ODTCS SAMs; 3:7 (A), 5:5 (B), 7:3 (C)

3. AFM images of deposited nanostructures

DNA triangle nanostructures were deposited on to the freshly prepared mixed and single component SAMs. By analyzing their AFM images of single component SAMs (*e.g.* APTES, BTCS, HTCS and ODTCS) in **Figure S4**, we conclude that the number density of adsorbed DNA nanostructure (per μm^2) increases with increasing the alkyl chain length of the silanes.

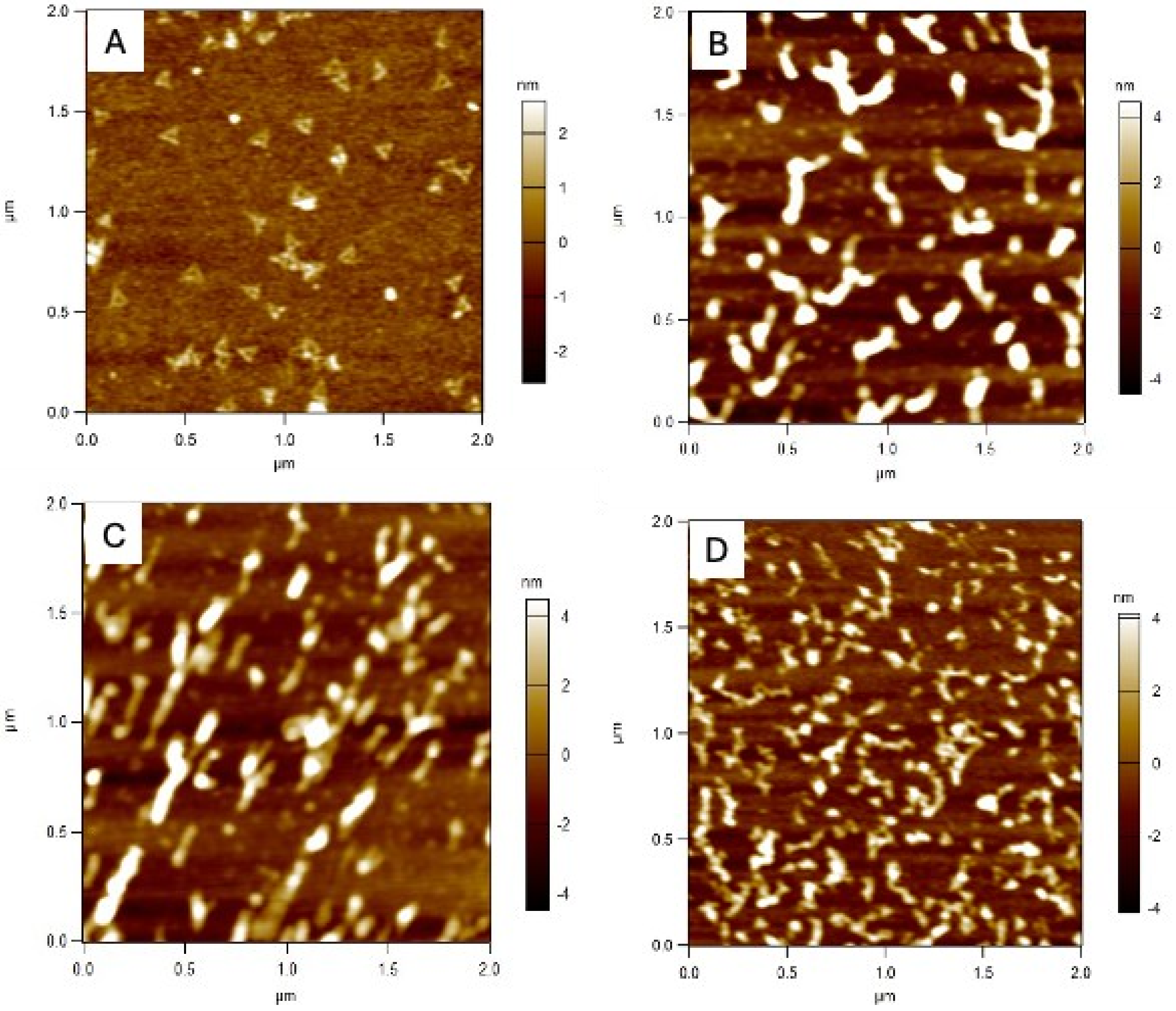

Figure S4. AFM images of DNA triangle nanostructures deposited on (A) APTES, (B) BTCS, (C) HTCS, and (D) ODTCS SAMs.

WCAs of mixed BTCS/APTES and HTCS/APTES SAMs showed only small changes (< 3 - 4°) as a function of their composition. DNA nanostructures deposited onto these surfaces were all deformed (**Figure S5, S6).**

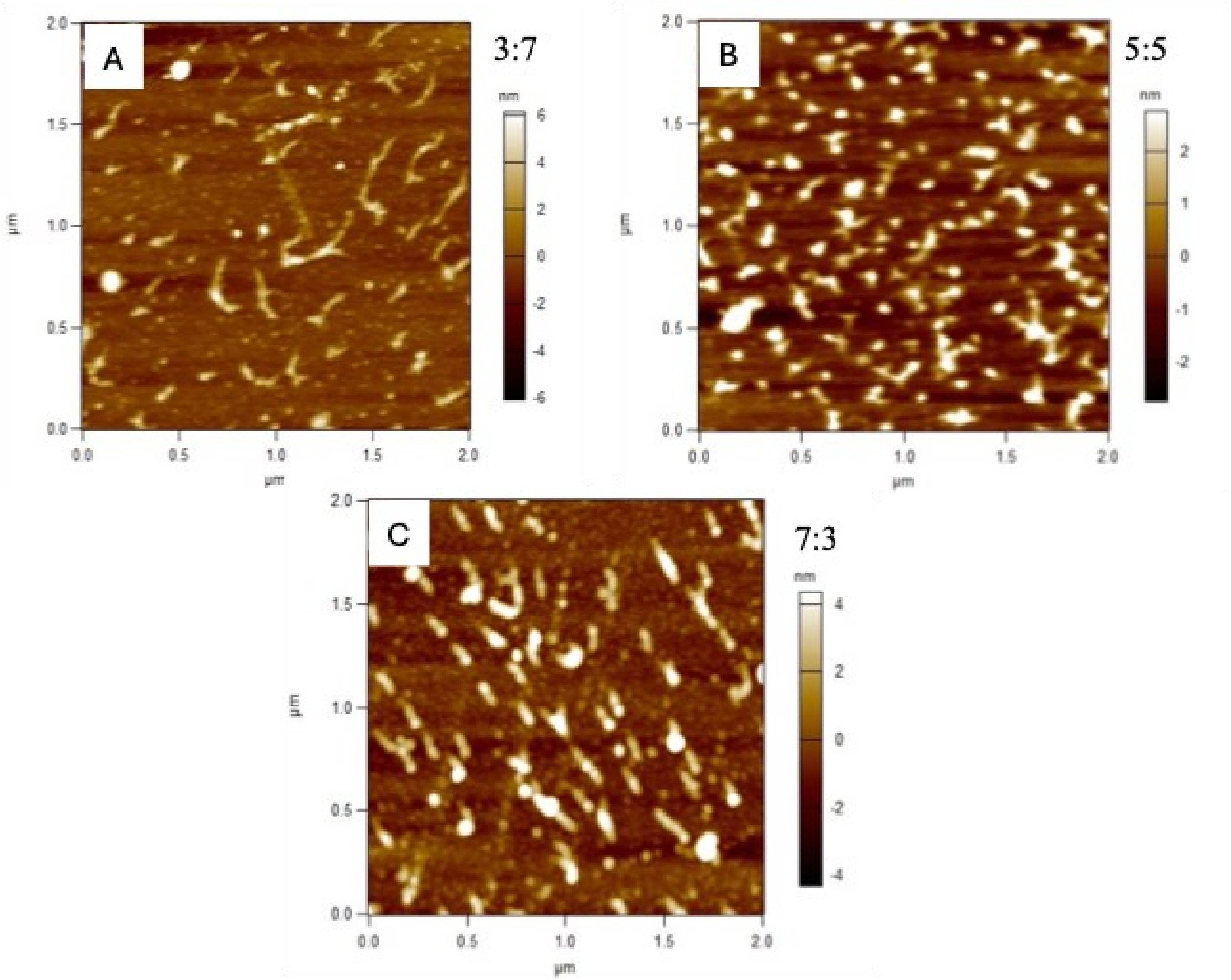

Figure S5. AFM images of DNA triangle nanostructures deposited on mixed BTCS: APTES SAMs; (A) 3:7, (B) 5:5, (C) 7:3

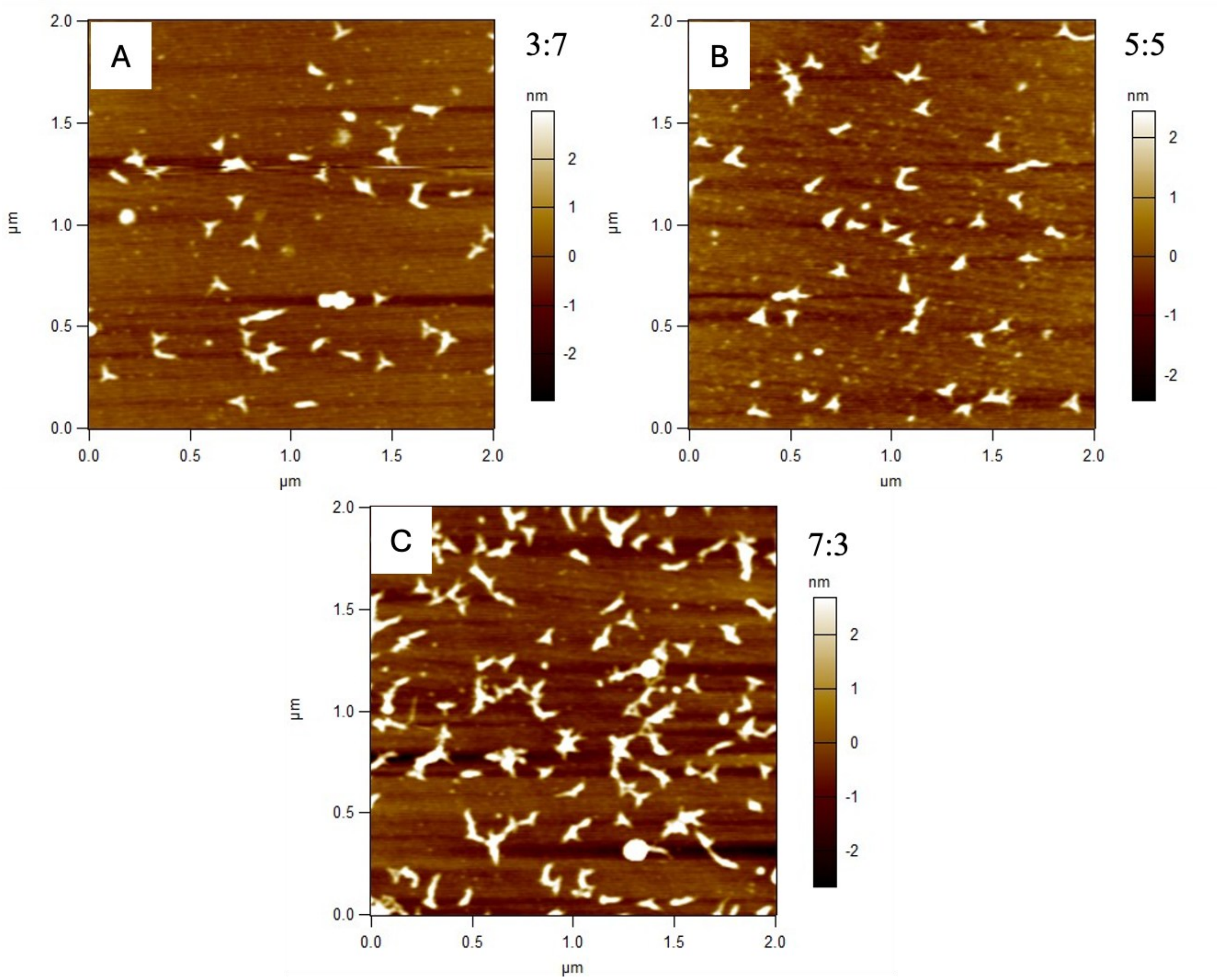

Figure S6. AFM images of DNA triangle nanostructures deposited on mixed HTCS: APTES SAMs of a ratio of: (A) 3:7, (B) 5:5, (c) 7:3

DNA nanostructures were imaged upon deposition onto Si wafer and mixed ODTCS: APTES SAMs with the mixed silanes ranging from 1:0 followed by 7:3, 5:5 and 3:7 to 0:1. As shown in the following figure, DNA nanostructures are stable on Si wafer and APTES SAM (**Figure S7 Blank** and **A**) and start deforming upon increasing the concentration of ODTCS in the silane mixture as shown in case of 7;3, 5:5 and 3:7. **Figure S7E** shows that nanostructures get completely stretched on single

component ODTCS SAM (0:1). Notably, number density of DNA nanostructures also increases with increasing the ODTCS concentration which is discussed further. AFM images obtained from a separate set of experiments are shown in **Figure S8**.

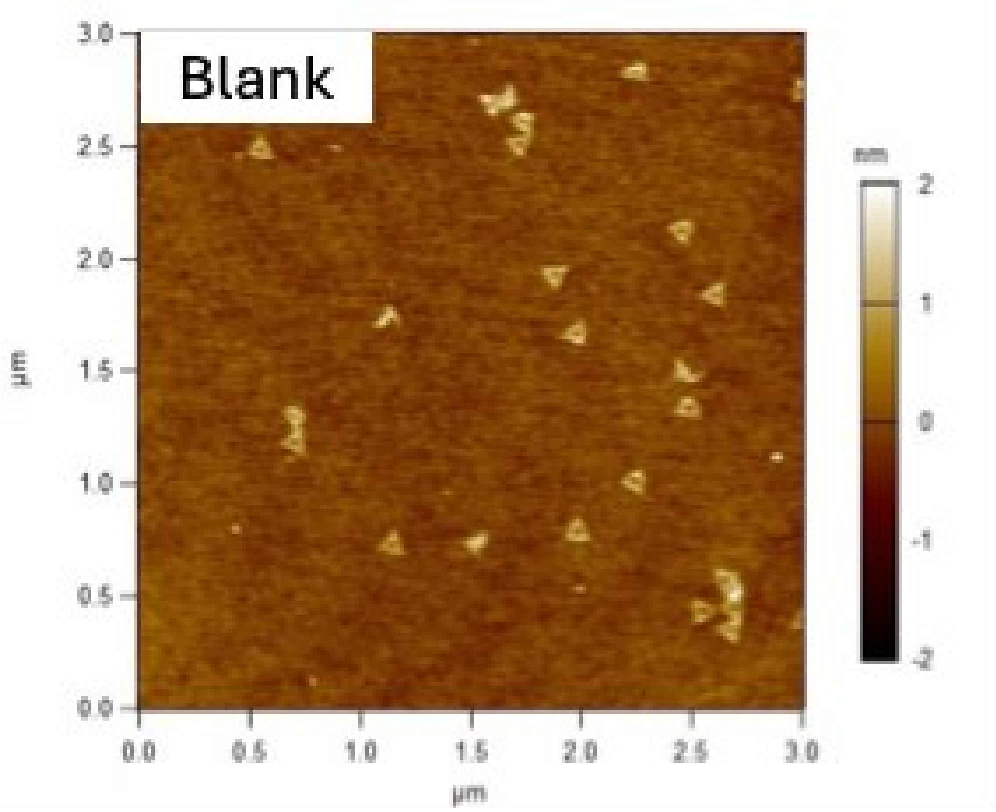

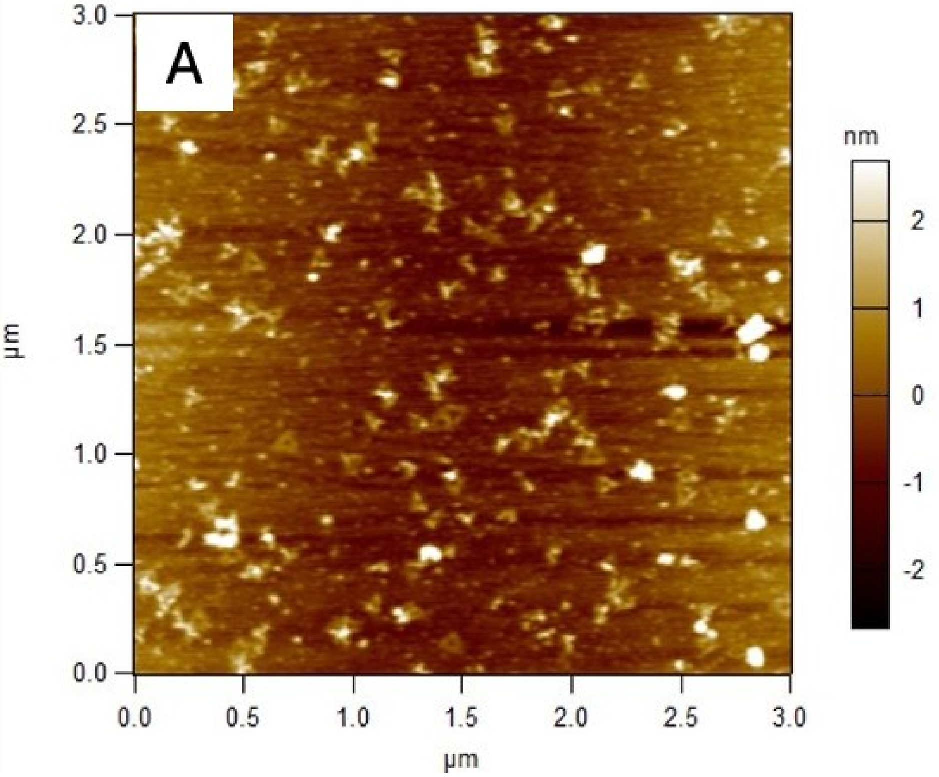

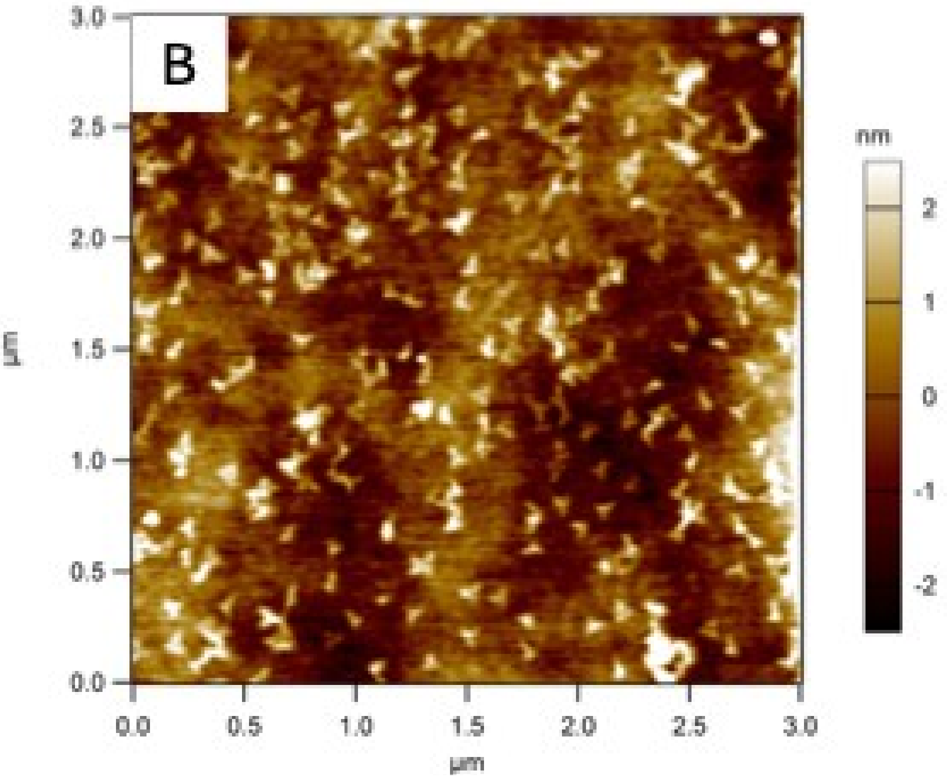
B
nm
2
1
0
-1
-2
µm
µm
0.0
0.5
1.0
1.5
2.0
2.5
3.0

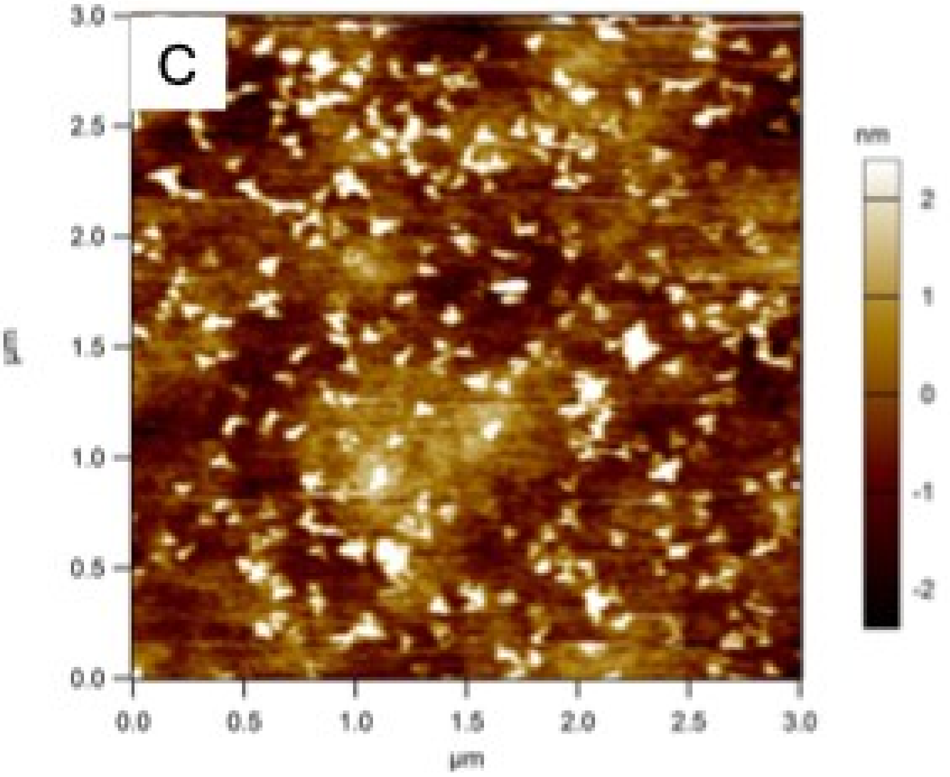
C
nm
2
1
0
-1
-2
µm
µm
0.0
0.5
1.0
1.5
2.0
2.5
3.0

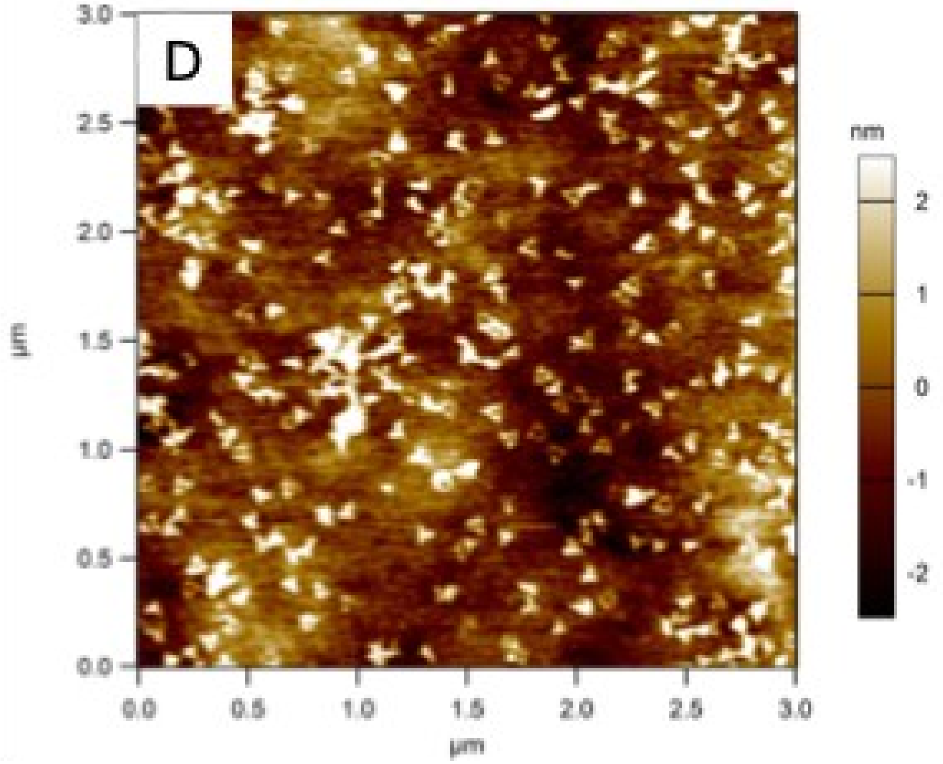

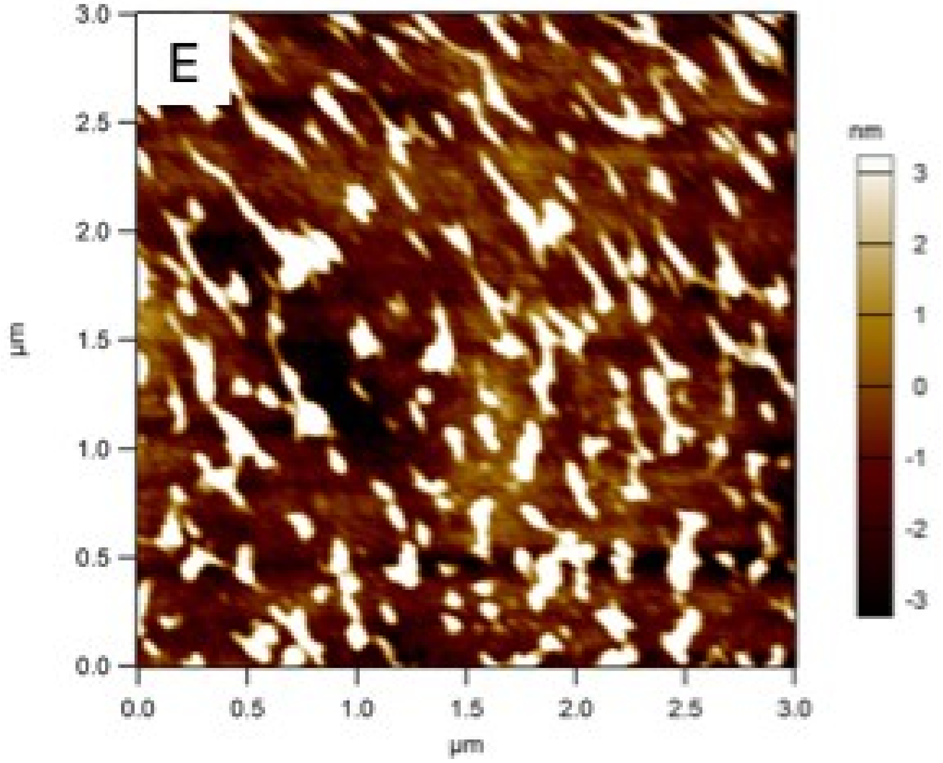

Figure S7. AFM images of DNA triangle nanostructures deposited on Si wafer (blank) and APTES: ODTCS mixed SAMs prepared with a ratio of silane precursor of (A) 1:0 (B) 7:3 (C) 5:5 (D) 3:7 (E) 0:1. This figure is the enlarged view of **Figure 1**.

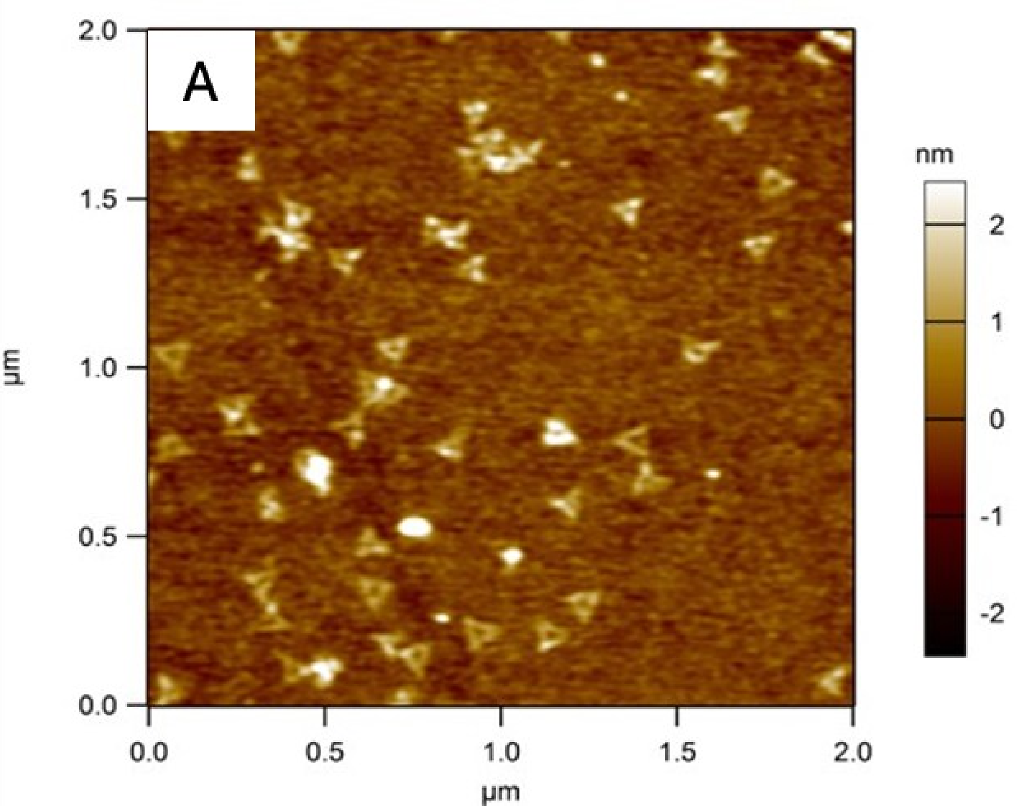
A
2.0
1.5
1.0
0.5
0.0
µm
0.0
0.5
1.0
1.5
2.0
µm
nm
2
1
0
-1
-2

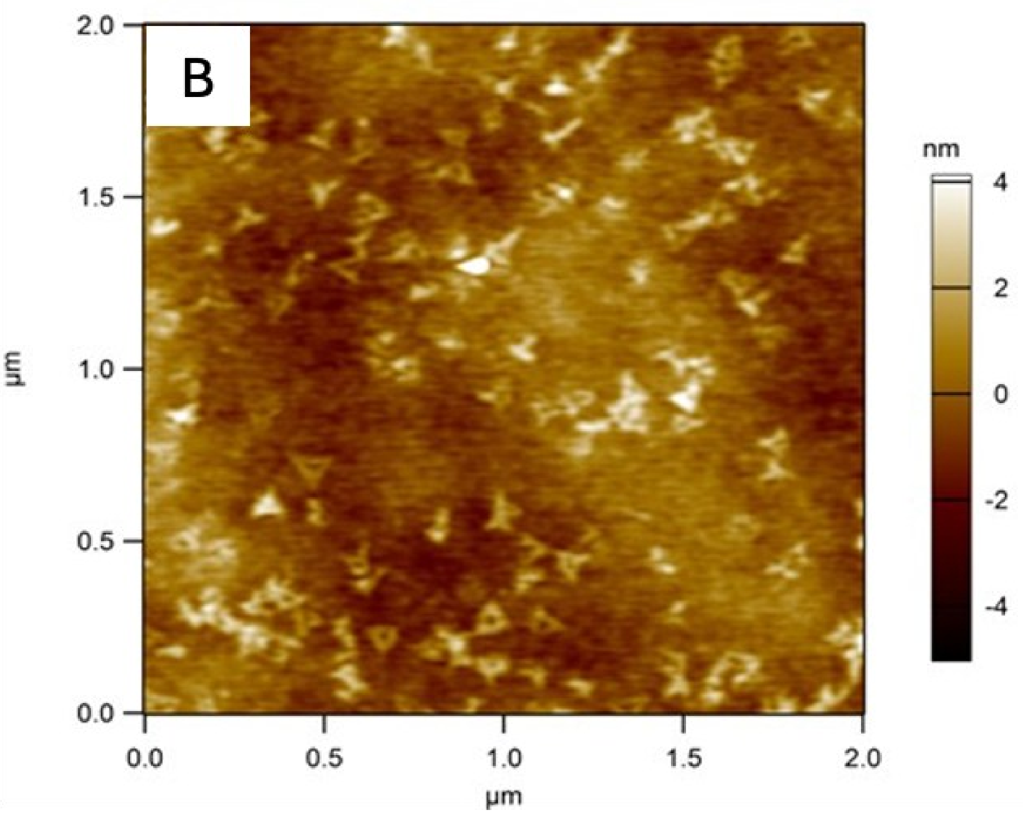
B
2.0
1.5
1.0
0.5
0.0
µm
0.0
0.5
1.0
1.5
2.0
µm
nm
4
2
0
-2
-4

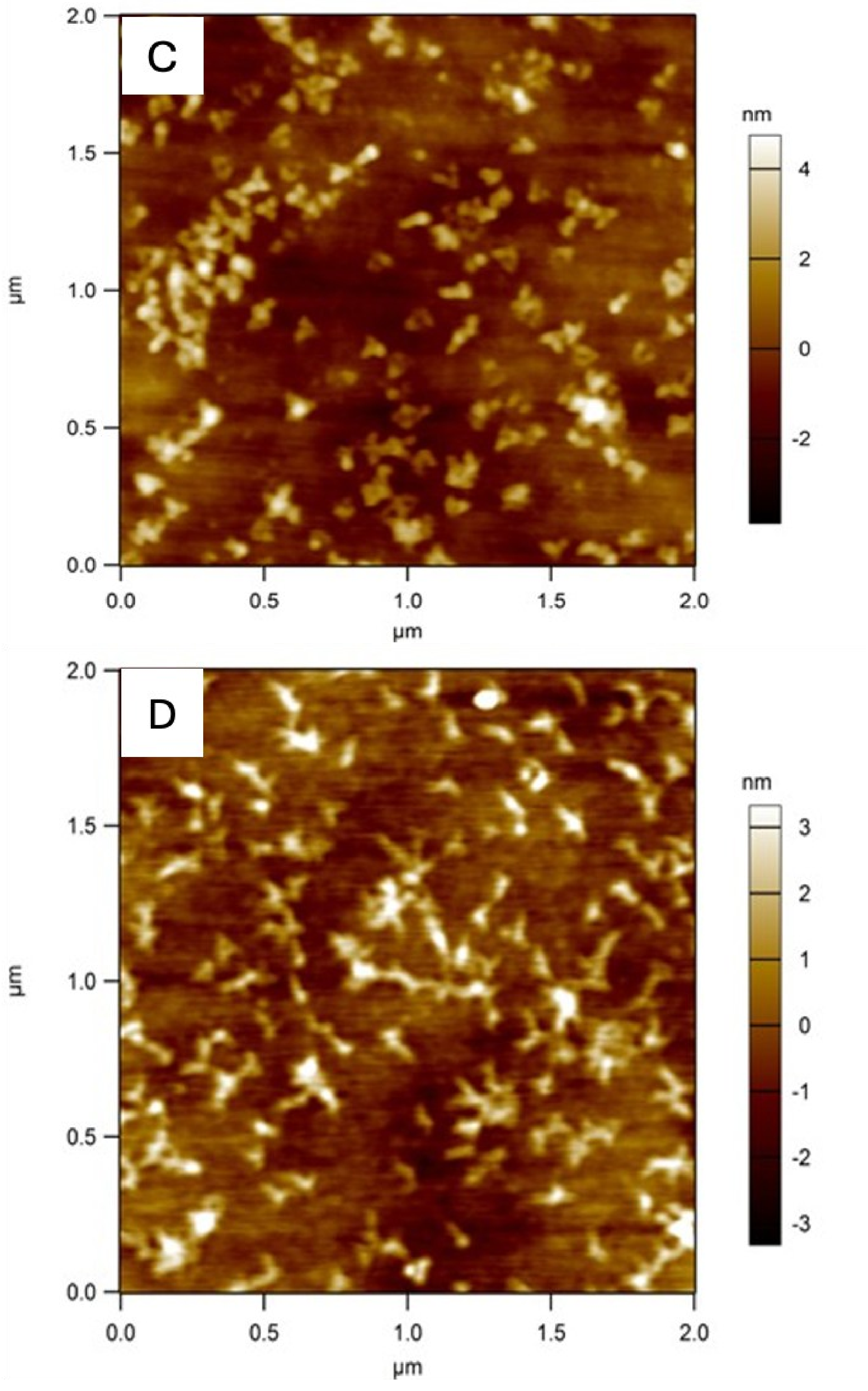
C
µm
0.0
0.5
1.0
1.5
2.0
nm
4
2
0
-2
D
µm
0.0
0.5
1.0
1.5
2.0
nm
3
2
1
0
-1
-2
-3

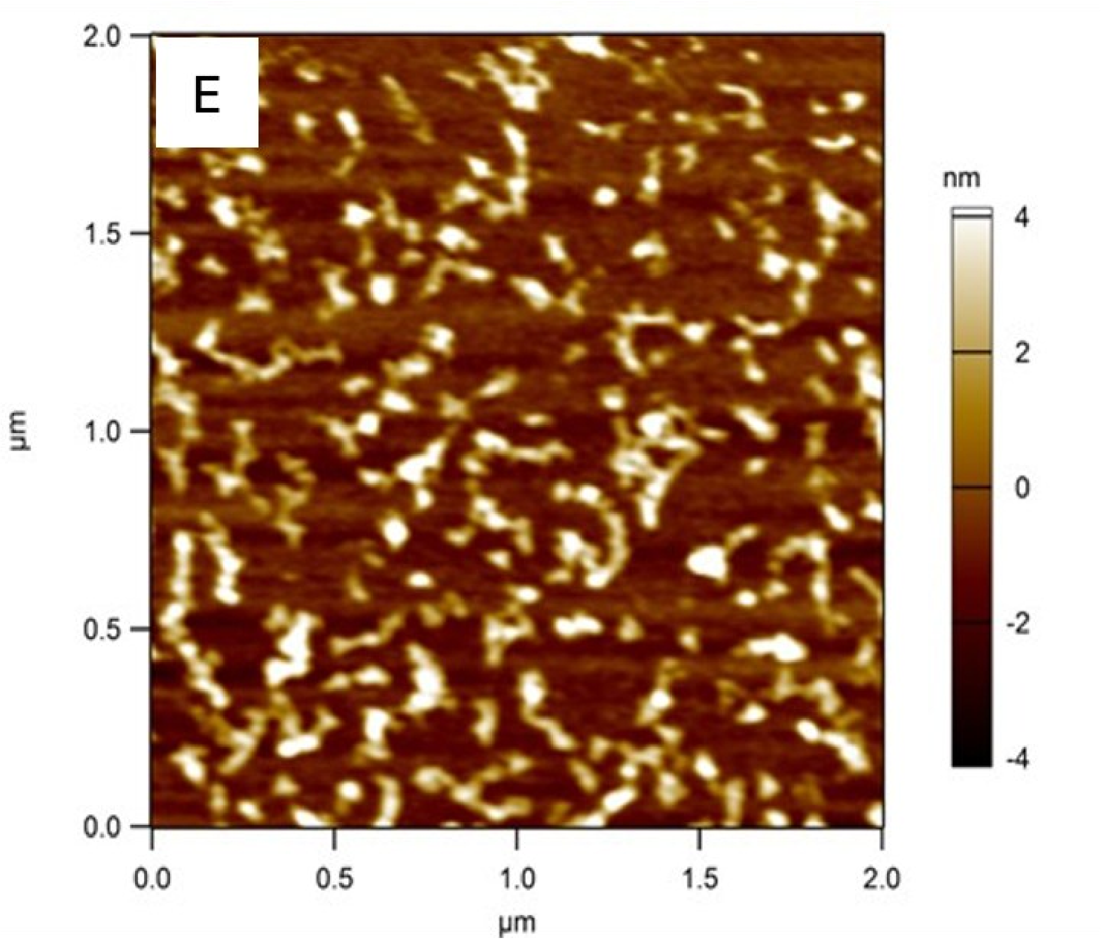

Figure S8. AFM images of DNA triangle nanostructures deposited on APTES: ODTCS mixed SAMs with a ratio of (A) 1:0 (B) 7:3 (C) 5:5 (D) 3:7 (E) 0:1. The data presented in this figure were obtained from a separate set of experiments than those shown in **Figure 1** and **S7**.

4. Morphology and number density of the DNA nanostructures deposited on SAMs

Table S1: Number density and percentage of intact DNA triangles (%T)

SAM Ratio [a]	10:0	7:3	5:5	3:7	0:10
Density [b]	56	34	36	34	11
%T [c]	0	15.2	26.4	39.6	62.2

[a] Molar ratio of the silanes (*i.e.* ODTCS: APTES) used in preparing the SAM. [b] Density: number of all DNA triangle nanostructure per μm^2. [c] %T: percentage of intact triangles (T)

Table S2. Morphology of DNA triangle nanostructures upon deposition onto single and mixed-SAMs

SAM	Nature of DNA triangles[a]				
Ratio[b]	0:1	3:7	5:5	7:3	1:0
ODTCS: APTES	T	PD+CD	PD+CD	PD+CD	CD
HTCS: APTES	T	CD	CD	CD	CD
BTCS: APTES	T	CD	CD	PD+CD	CD

[a]Nature of the DNA triangles: PD: partially deformed, CD: completely deformed, T: intact. [b]Molar ratio of the silane precursors used to prepare the SAM

5. References

S1. Rothemund, P. W. Folding DNA to create nanoscale shapes and patterns. *Nature* **2006**, *440* (7082), 297-302. DOI: 10.1038/nature04586.

S2. Hung, A. M.; Micheel, C. M.; Bozano, L. D.; Osterbur, L. W.; Wallraff, G. M.; Cha, J. N. Large-area spatially ordered arrays of gold nanoparticles directed by lithographically confined DNA origami. *Nature Nanotechnology* **2010**, *5* (2), 121-126. DOI: 10.1038/nnano.2009.450.

S3. Wang, Y.; Lieberman, M. Growth of ultrasmooth octadecyltrichlorosilane self-assembled monolayers on SiO_2. *Langmuir* **2003**, *19* (4), 1159-1167. DOI: 10.1021/la020697x.

S4. Tripp, C. P.; Hair, M. L. An infrared study of the reaction of octadecyltrichlorosilane with silica. *Langmuir* **1992**, *8* (4), 1120-1126. DOI: 10.1021/la00040a018.

S5. Flinn, D. H.; Guzonas, D. A.; Yoon, R. H. Characterization of silica surfaces hydrophobized by octadecyltrichlorosilane. *Colloids and Surfaces A: Physicochemical and Engineering Aspects* **1994**, *87* (3), 163-176. DOI: 10.1016/0927-7757(94)80065-0.

S6. Owens, D.K. and Wendt, R.C., Estimation of the surface free energy of polymers. *J. Appl. Polym. Sci.* **1969**, *13*, 1741-1747. DOI: 10.1002/app.1969.070130815.

S7. Kaelble, D. H.. Dispersion-polar surface tension properties of organic solids. *J. Adhes.* **1970**, *2*(2), 66–81. DOI: 10.1080/0021846708544582.
